# Estimating the malaria risk of African mosquito movement by air travel

**DOI:** 10.1186/1475-2875-5-57

**Published:** 2006-07-14

**Authors:** Andrew J Tatem, David J Rogers, Simon I Hay

**Affiliations:** 1 Spatial Ecology and Epidemiology Group, Tinbergen Building, Department of Zoology, University of Oxford, South Parks Road, Oxford, OX1 3PS, UK; 2 Malaria Public Health and Epidemiology Group, Centre for Geographic Medicine, KEMRI, P. O. Box 43640, 00100 GPO, Nairobi, Kenya

## Abstract

**Background:**

The expansion of global travel has resulted in the importation of African *Anopheles *mosquitoes, giving rise to cases of local malaria transmission. Here, cases of 'airport malaria' are used to quantify, using a combination of global climate and air traffic volume, where and when are the greatest risks of a *Plasmodium falciparum*-carrying mosquito being importated by air. This prioritises areas at risk of further airport malaria and possible importation or reemergence of the disease.

**Methods:**

Monthly data on climate at the World's major airports were combined with air traffic information and African malaria seasonality maps to identify, month-by-month, those existing and future air routes at greatest risk of African malaria-carrying mosquito importation and temporary establishment.

**Results:**

The location and timing of recorded airport malaria cases proved predictable using a combination of climate and air traffic data. Extending the analysis beyond the current air network architecture enabled identification of the airports and months with greatest climatic similarity to *P. falciparum *endemic regions of Africa within their principal transmission seasons, and therefore at risk should new aviation routes become operational.

**Conclusion:**

With the growth of long haul air travel from Africa, the identification of the seasonality and routes of mosquito importation is important in guiding effective aircraft disinsection and vector control. The recent and continued addition of air routes from Africa to more climatically similar regions than Europe will increase movement risks. The approach outlined here is capable of identifying when and where these risks are greatest.

## Background

Throughout history the opening of travel and trade routes between countries has been accompanied by the spread of diseases and their vectors [1,2]. Air travel has been identified as a prime factor in the global spread of both infectious and vector-borne diseases [3,4] and represents a longstanding concern [5]. Many recent disease vector invasions are suspected to have resulted from air travel [6-8] and, with continual rapid expansion in global air travel, the threat of future invasions should not be ignored. The public health and economic impacts of past disease vector invasions [2] are illustrated by *Aedes aegypti*'s invasion of the Americas [7] and the escape of *Anopheles gambiae *to Brazil from Africa [9].

The last 30 years has seen air travel to tropical regions of the World rise dramatically. International tourist arrivals to sub-Saharan Africa (SSA) increased from 6.7 million in 1990 to over 17 million in 2000 [10]. Mosquito species, including *Aedes albopictus *and *An. gambiae *s.l., have been shown to survive long haul flights [11-13]. For example, in one 3-week period in 1994, it was estimated that 2000–5000 *Anopheline *mosquitoes were imported into France at a rate of 8–20 *Anopheline *mosquitoes per flight [6]. An example of the effects of such importations are the many cases of autochthonous (locally-acquired) malaria, which are principally clustered around international airports: so called "airport malaria" [14,15]. These occur primarily through the transport of infected *Anopheles *mosquitoes that can survive for long enough after arrival to transmit malaria [14]. Recent studies suggest that, where used, routine disinsection is proving effective in reducing airport malaria risk [16], although the number of countries implementing such procedures is in decline [17,18]

Airport malaria cases are rare, with just two cases per year on average recorded (1969–99), all but one of which were *P. falciparum *malaria. However, these cases provide important evidence of sufficient traffic volumes and climatic similarity between origin and destination for the survival of malaria-carrying *Anopheles *mosquitoes. Here we describe an exploratory approach which makes use of this information derived from airport malaria cases to quantify, in terms of global climate and air traffic, which airports have the greatest risks of local *P. falciparum *malaria transmission through importation from sub-Saharan Africa of infected mosquitoes. We estimate a) the risks based on year 2000 air traffic volumes, b) how this varies throughout the year and c) where the greatest potential future risks would lie through the opening of new routes.

## Methods

### Data

Flight data on total passenger numbers in the year 2000 moving between the World's top 100 airports by traffic (100% aircraft capacity was assumed), were obtained from OAG Worldwide Ltd [19]. The database includes cargo flights. For full geographical coverage, the database also included data on the principal airports of 143 other countries not represented in this top 100. Data on a total of 7129 routes between 278 international airports in the year 2000 were thus available. To provide an estimate of the most likely months of malaria movement, information on the onset and end of the principal malaria transmission seasons for each African country were obtained from maps of malaria seasonality across Africa [20]. These maps were derived only for *P. falciparum *transmission by *An. gambiae *s.l., and all results presented here refer only to this parasite and vector combination. The results may be extended to other species, but this paper concerns itself with the most efficient vector of malaria (*An. gambiae*) and the most pathogenic of the malaria parasites (*P. falciparum*) [21,22].

### Climate signatures

A 10 × 10 minute (~18 × 18 km at the equator) spatial resolution gridded climatology was used to extract mean temperature, rainfall and humidity data for a synoptic year (1961–1990) [23], and these layers were then linearly rescaled to a common range of values by dividing through by the maximum in each layer. Climatic similarity between origin and destination airports was assumed to determine principally whether mosquitoes from originating airports would survive and have the potential to produce local malaria transmission around the destination airports. The locations of the 278 airports were superimposed onto the monthly climatology surfaces and each 10 × 10 minute spatial resolution grid square covering the airport location identified. To ensure a representative climate was included, the eight land grid squares surrounding each airport square were also identified, forming a three by three grid square centered on the airport. This was not possible for coastal airports or those on small islands, where reduced numbers of land grid squares were extracted. Airports located on islands too small to be represented by the climatology surfaces were eliminated from the analysis, reducing the sample size to 259 airports. Ideally, data for these airports would have been included in the analysis, but the need to use a global climatology meant this was not possible. Data from the grid squares identified in each of the three climatology surfaces thus formed climate 'signatures' for each month, for each airport.

### Distance measures, clustering and dendrograms

The lack of sufficient variance in the climate data constituting the majority of signatures, and the location of many airports on small islands, dictated that only simple Euclidean distance could be used as a measure of climatic similarities between origin and destination airports [24]. Euclidean distance is defined as the shortest straight line distance between two points, in this case, the distance between the environmental signature centroids in three-dimensional climatic space, as defined by the temperature, rainfall and humidity values. Euclidean distances between each signature centroid and the centroids of every other signature were calculated to derive separate monthly climate "dissimilarity" matrices. The climate dissimilarity matrices were then subject to hierarchical clustering using an agglomerative algorithm. The clustering results were then translated into dendrograms based on centroid linkage using Phylip v3.63 (University of Washington, USA), for each month of the year. The dendrograms are monthly climate-based phenetic trees, and represent the global air transport network remapped in terms of disease vector suitability [2,24]. Tests using those signatures which did facilitate the use of more sophisticated distance measures (e.g. divergence, Jefferies-Matusita), revealed that the resultant dendrogram architecture was very similar to those developed using Euclidean distances (results not shown).

### Climatic similarity thresholds

To define how similar airport climates need to be to permit the temporary survival of imported *Anopheles *and the possible transmission of *P. falciparum *malaria, confirmed examples of where this has occurred were used. These cases of airport malaria confirm that, at the time of year of the case, the climates in the vicinity of the origin and destination airports were similar enough for the survival of malaria-carrying *Anopheles*, and transmission by them at the destination. In no suspected cases of airport malaria has the origin of imported *Anopheles *or malaria been confirmed unambiguously, so it was assumed that the origin was the SSA airport or region most climatically similar to the destination and within its primary transmission season. This gives the most conservative estimate of the climatic range within which imported malaria transmission is possible.

**Figure 1:**
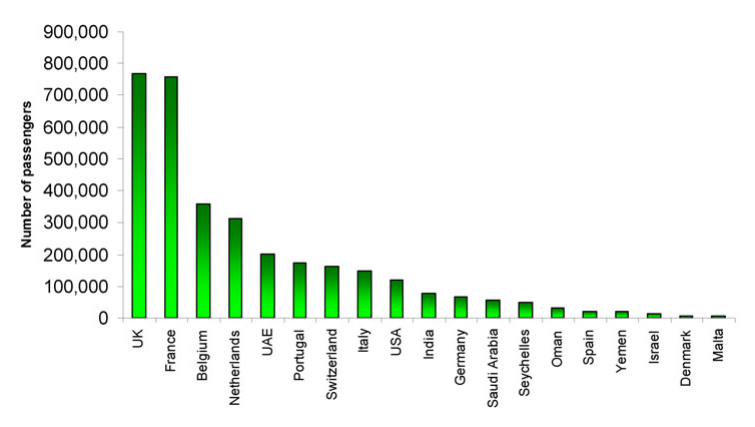
Total incoming passenger numbers per country from malaria endemic African airports for 2000.

**Figure 2:**
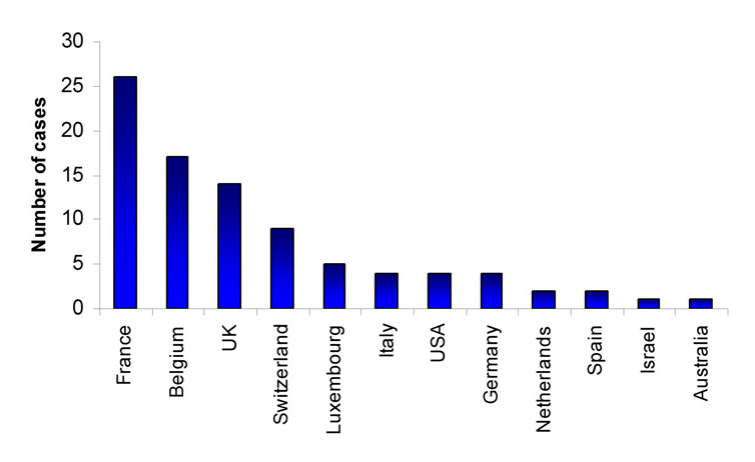
Countries in which confirmed or probable cases of airport malaria have been reported. Data taken from Gratz (2000)[6].

**Figure 3:**
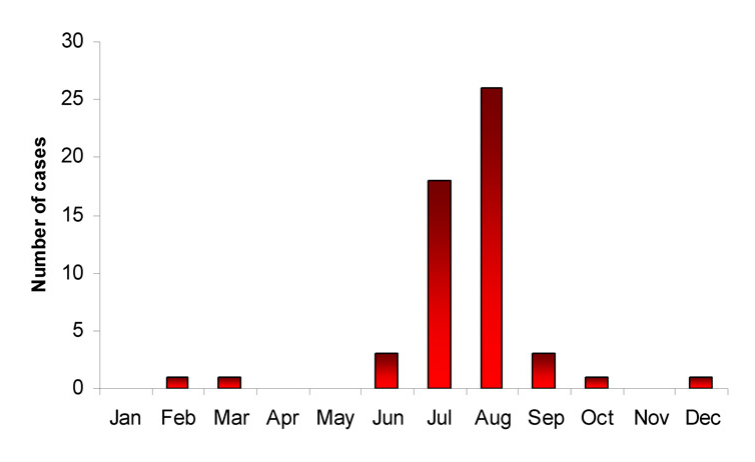
Month in which European airport malaria cases occurred [7, 8, 14, 25, 38-62] (where date is provided).

Figure 1 shows incoming passenger volumes from malaria-endemic African airports for 2000, where suspected cases of airport malaria have been reported from 1969–1999 and, where details were provided, the month of European airport malaria cases. Figures 2 and 3 show that in the last 30 years the vast majority of probable airport malaria cases have occurred in the months of July and August, in the vicinity of Paris Charles De Gaulle airport, London Heathrow and Gatwick airports and Brussels airport [6]. All routes linking these destination airports to departure airports in malaria endemic SSA countries with transmission in July and August were identified. The routes were located on the relevant climatic dendrograms and the branch height joining the most similar origin and destination airports in question noted. The height of the highest branch joining airport malaria origin to destination airport was then taken as the climatic similarity limit for temporary *Anopheles *survival, once imported. This provided an empirically-defined conservative climatic tolerance limit in terms of temperature, rainfall and humidity. This limit was then applied across all dendrograms.

The African airports in the database were overlaid on the malaria seasonality maps and classified as either suitable or not for transmission during each month of the year. Those non-malarious airports linked *via *a dendrogram branch lower than the airport malaria-defined climatic limit to a malarious airport during its transmission season were identified as being sufficiently similar climatically, based on previous airport malaria cases, for there to be a risk of infectious mosquito arrival, survival and possible consequent local malaria transmission.

### Risk matrices

To obtain a monthly measure of malarial mosquito movement risk to those airports identified as being at risk within the dendrogram in 2000, the monthly Euclidean environmental distances and traffic data were used. The Euclidean distances between malarious airports and other airports were rescaled linearly by dividing through by the maximum Euclidean distance in the climate dissimilarity matrix, and inverted to lie between zero and one. Therefore, the most climatically similar airport pairs had a value close to one and those distinctly different, a value close to zero. Similarly, the air traffic data were also rescaled, resulting in the airports with the most direct traffic running between them in a year taking a value close to one, while those direct routes with little or no traffic a value close to or at zero. It was assumed for this analysis that passenger numbers on each route remained consistent year-round. For each month and route between malarious and other airports, the rescaled climatic and traffic matrices were then multiplied together to provide a simple monthly measure of the likelihood of temporary malarious mosquito importation and possible consequent local transmission.

### Sensitivity of results

The results obtained are dependent upon both the accuracy of the seasonality map and the choice of dendrogram threshold. It was therefore essential to assess how sensitive these results were to small alterations in each. The approach, results and related discussion are provided in Additional File 1, and demonstrate that the results presented remain relatively insensitive to small changes in the seasonality map and choice of dendrogram threshold.

## Results

### Year 2000 situation

Table 1 shows the top ten air travel risk routes for malaria-carrying *Anopheles *invasion in operation in 2000 based on dendrogram-thresholding, combined air traffic volumes and climatic similarity between departure and destination airports. All ten routes fly to European destinations in July, August or September. Nineteen risk routes were identified in total, with all (n = 8) destination airports having previously reported cases of local *P. falciparum *transmission.

**Table 1:** Year 2000 top 10 air travel risk routes for *P. falciparum *infected *An. gambiae *invasion and subsequent autochthonous transmission.

	From		To			
Rank	Airport	Country	Airport	Country	Month	Annual No. Passengers

1	Abidjan	Cote d'Ivoire	Paris Charles de Gaulle	France	August	169,188
2	Accra	Ghana	Amsterdam Schippol	Netherlands	July	53,130
3	Entebbe/Kampala	Uganda	Brussels	Belgium	July	42,141
4	Accra	Ghana	Amsterdam Schippol	Netherlands	September	53,130
5	Abidjan	Cote d'Ivoire	Brussels	Belgium	August	58,021
6	Accra	Ghana	Rome Fiumicino	Italy	September	12,420
7	Abidjan	Cote d'Ivoire	Zurich	Switzerland	July	46,495
8	Accra	Ghana	Rome Fiumicino	Italy	August	12,420
9	Abidjan	Cote d'Ivoire	London Gatwick	United Kingdom	August	37,843
10	Cotonou	Benin	Brussels	Belgium	August	14,954

### Future risks

**Figure 4:**
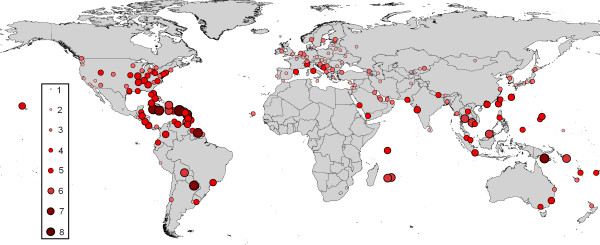
Number of months in a year that the climate at each international airport is sufficiently similar to that of a SSA airport within its primary malaria transmission season for imported *P. falciparum*-carrying *An. gambiae *survival.

**Figure 5:**
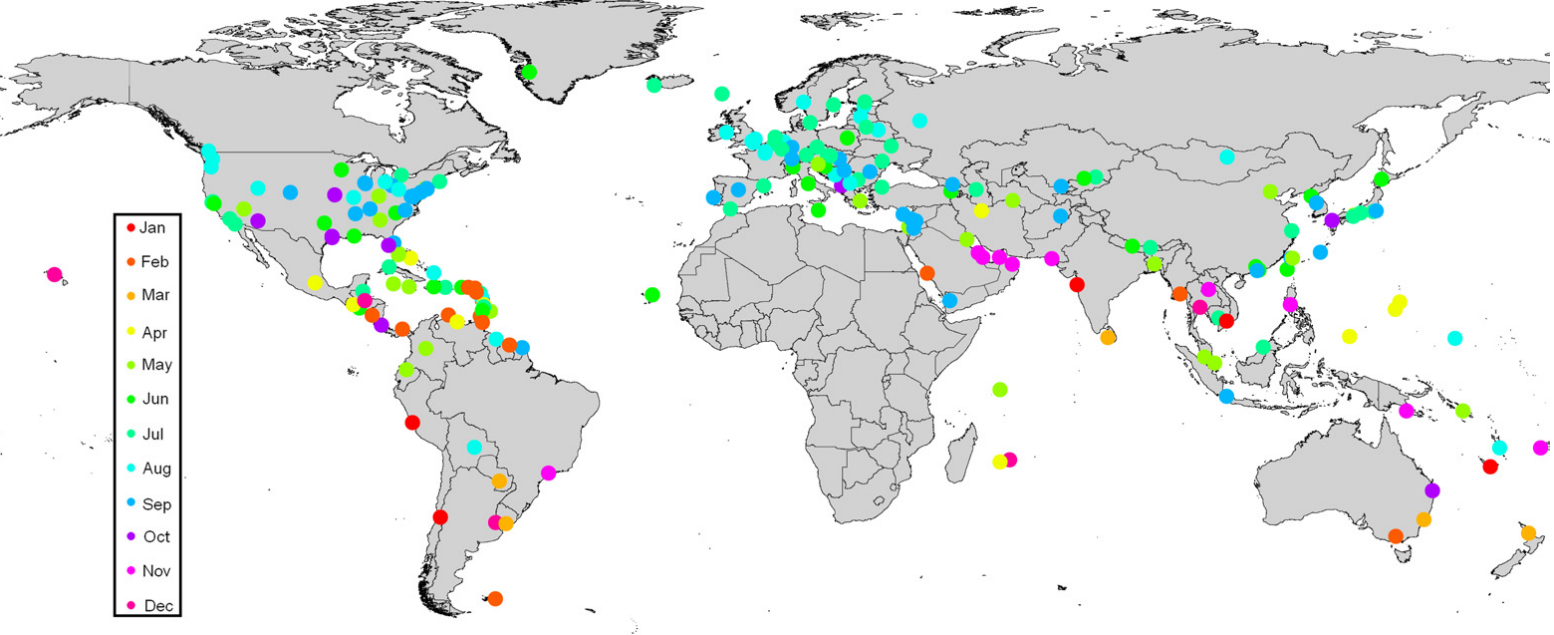
Month of peak climatic similarity with climatically closest malaria-endemic SSA airport.

Examination of the monthly climate dissimilarity matrices between SSA airports within principal malaria transmission season and all other airports globally, allowed identification of the most climatically similar airports through the year. Table 2 shows the top twenty airport pairs representing the greatest risks of imported *P. falciparum*-carrying *Anopheles *survival, should these routes become operational in the future. Utilisation of the airport malaria thresholded vector-movement dendrogram allows for the examination of the number of months per year that the climate at each airport is sufficiently similar to its nearest malarious SSA airport climatically for imported *P. falciparum*-carrying *Anopheles *survival. These results are shown in Figure 4. Figure 5 shows the specific month when the destination airport climate is closest to the malarious origin airport.

**Table 2:** Top 20 climatically closest linked destination airports with malarious-SSA airports within principal transmission season per-month.

	From		To		
Rank	Airport	Country	Airport	Country	Month

1	Conakry	Guinea	Bangkok	Thailand	Dec
2	Manzini	Swaziland	Brisbane	Australia	Oct
3	Conakry	Guinea	Managua	Nicaragua	Feb
4	Dar Es Salaam	Tanzania	Fort Lauderdale	USA	May
5	Maputo	Mozambique	Paramaribo	Surinam	Feb
6	Ndjamena	Chad	Santo Domingo	Dominica	Jul
7	Lusaka	Zambia	Guatemala City	Guatemala	Apr
8	Lome	Togo	St Vincent	St Vincent and the Grenadines	June
9	Dar Es Salaam	Tanzania	Miami	USA	May
10	Entebbe	Uganda	Detroit	USA	Aug
11	Accra	Ghana	Grand Cayman	Cayman Islands	May
12	Accra	Ghana	Montego Bay	Jamaica	May
13	Pointe Noire	Congo	Sal	Cape Verde	June
14	Ouagadougou	Burkina Faso	Havana	Cuba	Jul
15	Entebbe	Uganda	Nashville	USA	Sep
16	Maputo	Mozambique	Tampa	USA	Oct
17	Dar Es Salaam	Tanzania	Asuncion	Paraguay	Mar
18	Lilongwe	Malawi	Caracas	Venezuela	Apr
19	Ndjamena	Chad	Jakarta	Indonesia	Sep
20	Abidjan	Cote d'Ivoire	Miami	USA	Oct

## Discussion

### Year 2000 situation

The routes identified in table 1 as the most likely for temporary malarious mosquito invasion and possible consequent autochthonous transmission in 2000 reflect both the structure of the global air traffic network and sufficient climatic similarities between SSA and Europe in July, August and September. There is strong correspondence between the risk routes and suspected airport malaria case location (Figure 2) and timing (Figure 3). The predominance of West African airports in table 1 occurs because the malaria transmission season timing coincides with the European summer. Although many other airports around the World were identified as more similar climatically to malarious SSA airports than the airport malaria examples, the majority of flights originating from SSA have European destinations, with over 100,000 per annum travelling on certain routes (e.g. Abidjan to Paris, see Table 1). Figure 4 shows that European destinations may be sufficiently similar climatically to SSA airports for malarious mosquito survival only 2–4 months a year, yet because almost all air traffic is directed toward Europe, the continent receives the majority of imported *P. falciparum *cases [25] and almost all recorded airport malaria episodes (Figure 1) [6]. Such a short window of sufficient climatic similarity suggests that increased vigilance and disinsection in terms of malaria-carrying *Anopheles *importation by incoming flights from SSA may only be required during the summer months in Europe, especially during unusually hot and humid periods. Disinsection, however, is also undertaken for arbovirus-carrying mosquito control. This paper has not addressed this risk, but other work suggests shipping to be most important in their spread [7,24].

The likelihood of *P. falciparum *or *An. gambiae *establishment through air travel in Europe and many other locations remains low. Unsuitable year-round climate, enforced disinsection measures, *An. gambiaes*' intolerance of urban areas [26] and competition from local mosquitoes that are inefficient vectors of *P. falciparum *all provide barriers to establishment. However, that all destinations identified for 2000 have experienced local malaria transmission within the months shown, is evidence of the existing risks and the need to incorporate climatic information in predictions of future risks. Although a conservative climatic similarity threshold was chosen, the fact that only 19 risk routes (of a possible 1,032) were identified for 2000, shows that the current air traffic network is restricted climatically, with almost all air traffic from SSA directed to climatically dissimilar European airports. This is perhaps one of the reasons why *An. gambiae *and *P. falciparum *malaria have escaped so few times from Africa.

Evidence of the importance of shipping traffic volume, particularly container shipping, in vector invasion has been outlined elsewhere [2,24]. While the focus of this analysis has been on passenger flights, this includes thousands of cargo-laden flights, and in terms of cargo-only flights, database interrogation shows that these make up less than 7% of all scheduled flights. The role of passenger air traffic volume is emphasized here through comparison of Figures 1 and 2. The spatial pattern of airport malaria cases is reflected in the annual incoming passenger numbers, with the adjusted r^2 ^= 0.704 (n = 19, p < 0.01) between the two. The fact that France has received more suspected cases of airport malaria than the UK, despite similar incoming passenger volumes from endemic airports, can be attributed to more routes linking it to West Africa, where transmission season and European summer timings match, though other factors, such as differences in surveillance systems, may play a part. Given that air traffic volumes generally peak in the northern hemisphere summer months, it should be additionally noted that the year round constant traffic volumes assumed here could produce an underestimation of risk levels.

### Future risks

The results show that many airports in regions outside of Europe are climatically more favorable than is Europe for *Anopheles *survival, and for many more months of the year. The effects of opening up new routes from malaria-endemic SSA countries to non-European destinations could therefore have potentially serious consequences. The accidental introduction of *An. gambiae *into Brazil in 1930, resulted in over 16,000 malaria deaths and a mosquito control program that cost 3 billion USD equivalent today [9,27]. Recently, new routes have been opening, with long-haul flights from SSA direct to Washington, Beijing, Hong Kong and Bangkok amongst others. With more routes planned, and larger planes capable of traveling greater distances in shorter times scheduled, the risk of another *An. gambiae *escape grows [28]. While recent evidence points to the effectiveness of routine aircraft cabin disinsection in flights to the UK from SSA [16], elsewhere in the World disinsection is in decline. The World Health Organization continues to recommend aircraft disinsection [29], but fears over the health effects of the pyrethroid insecticides used [30] and resultant law suits [31] have led to many airlines and governments ceasing the practice [17,18].

The destinations in Table 2 with climates matched almost perfectly for the month in question to SSA airports home to *P. falciparum *infected *Anopheles *are all in malarious regions, or those declared malaria-free only recently [32,33]. Should regular flights commence on any of those routes identified in Table 2, the potential for temporary *An. gambiae *invasion and consequent *P. falciparum *transmission in those months may be considerable. Though many in Table 2 appear to be unlikely routes in the near future, just outside the top 20 are routes of potential future operation or routes which have recently opened (e.g. Addis Ababa to Washington, Dakar to New York). A route for almost every month of the year is identified in the top 20 alone, highlighting that should SSA become better connected by air, a year-round linkage with climatically similar airports would exist, potentially facilitating the escape of *An. gambiae *from SSA. Moreover, the opening of flight routes from south-east Asia to SSA may also speed up the global movement of drug resistant malaria (in humans or mosquitoes) and insecticide resistant mosquitoes [34,35].

Figure 4 shows, as expected, that the climatic regimes of the airports in the tropical regions of the Americas and south-east Asia are more similar to those of malaria-endemic SSA airports than are the latter to those of most European airports. This is especially true of the airports in Central America and the Caribbean which have the closest match of all airports globally, and for the longest period. This explains the predominance of Caribbean and Central American destination airports in Table 2.

Unsurprisingly the periods of greatest similarity of higher latitude climates to those of SSA airports are the summer months of May to September in the northern hemisphere and November to February in the southern hemisphere (Figure 5); it is, therefore, during these months that airport malaria in these regions is most likely. The equivalent periods of similarity for tropical latitudes are much more variable, with little apparent spatial coherency in peak month. Evidence of this can be seen in Table 2, which includes two routes to Miami, USA, one from Dar Es Salaam in East Africa in May, the other from Abidjan in West Africa in October. In the past, high risk locations and months for temporary *Anopheles *invasion leading to local transmission have been predictably along specific routes to certain European airports, and solely in the summer months. Figure 5 shows that should routes between *P. falciparum*-endemic SSA countries and other tropical locations continue to open up, the risks, origin and timing of *Anopheles *invasion will be much less predictable.

## Conclusion

The continued occurrence of airport malaria cases and rise in imported *P. falciparum *malaria cases [15,25] are indicative of an expanding global air transport network and increased travel to malarious countries [36]. It also reflects a decline in aircraft disinsection [17,18]. The approach presented highlights routes and months within the current global air transport network at risk of importation and temporary establishment of *P. falciparum*-carrying mosquitoes. The development of new air travel routes from SSA suggests that the relative risks identified will continue to increase and that monitoring schemes based on climate suitability methods could be used to optimize *Anopheles*-specific disinsection and control efforts. The analysis presented here is a first step, and future challenges to refine the predictions will involve incorporating information on temporal variations in passenger numbers, flight stopover risks, intra-species competition, human populations at risk, breeding site availability, possible climate change [37], disinsection and land transport, and quantifying the relative importance of sea and air transport for vectors and diseases.

## Authors' contributions

AJT conceived, designed and implemented the research and wrote the paper. DJR provided methodological and editorial input. SIH helped conceive the research and aided in the paper writing and editing. All authors have read and approved the final manuscript.

## Supplementary Material

Additional File 1 A supplementary file is included: which describes the methodology, results and discussion relating to the sensitivity analysis. Click here for file
