# Supplementary material for: Estimating the malaria risk of African mosquito movement by air travel
Source: Malar J. 2006 Jul 14;5:57. doi: 10.1186/1475-2875-5-57 (PMC1557515; doi:10.1186/1475-2875-5-57)
Supplement: Additional File 1 — A supplementary file is included: which describes the methodology, results and discussion relating to the sensitivity analysis. [file 1475-2875-5-57-S1.doc]

**Additional File 1**

***Sensitivity of results***

The results obtained are dependent upon both the accuracy of the seasonality map and the choice of dendrogram threshold. It was, therefore, essential to assess how sensitive these results were to small alterations in each.

**Methods**

Four new African malaria seasonality maps were created by shifting the malaria season start both forward and backward by one month (where possible) and applying the same shifts to the season end map. These new maps were then used, with the original map, to recalculate the results for all possible combinations of maps. Results of the effects of these seasonal shifts are shown in Table 1.1.

The sensitivity of the dendrogram threshold selection criteria was also tested. The lack of definitive information on airport malaria case origins meant that the results presented in this paper were based on a dendrogram threshold defined by the most climatically similar SSA airport (within its primary malaria transmission season) linked to Paris, London or Brussels airports in July and August. Should this conservative threshold choice be incorrect, the effect on results, through determining the dendrogram threshold defined by the most climatically *dissimilar* SSA airport linked to Paris, London or Brussels airports in July and August, was examined (criterion ‘a’). In addition, the effects of choosing the threshold based on the most climatically similar SSA airport for both *June* (criterion ‘b’) and *September* (criterion ‘c’) were examined. Results of the effects of these alternative dendrogram thresholds on presented results are shown in table 2.

**Results and Discussion**

The simple sensitivity assessments allow the effects of uncertainties in the input data and assumptions made to be examined. Table 1.1 shows that, in general, the results produced from this study are relatively insensitive to any small errors that may exist in the seasonality map. Many risk routes in Tables 1 and 2 of the main paper have year-round transmission and so are not affected by seasonal shifts (though the intensity of transmission is likely to vary seasonally). The remaining routes have long seasons of transmission in SSA and are linked with destinations outside Europe that are similar climatically in the middle of their seasons. No obvious spatial pattern was evident in terms of the small percentage of airports in Figures 2 and 3 that changed.

Table 1.2 shows that the choice of dendrogram threshold criteria has more of an influence on results, but that they remain relatively insensitive to small alterations. The results from criterion ‘a’ demonstrate that, should the assumption of previous airport malaria cases originating from the most closely linked SSA airport climatically be incorrect, the results presented here would be relatively unaffected. The similarity of climates, and consequent clustering of SSA airports within their principal malaria transmission seasons on each monthly dendrogram, particularly in the July-September period, means that there is very little difference in most cases between defining thresholds using the most climatically similar and dissimilar SSA airport branch link to London, Paris or Brussels airports. This clustering of SSA airports on the dendrograms and climatic similarities to airports elsewhere in June and September means that dendrogram thresholds defined by criteria ‘b’ and ‘c’ also, in general, have little effect on results. Some changes are seen in these months, however, as climatic similarity decreases across the northern hemisphere, where the majority of airports are located. The largest changes are produced using criteria ‘b’ for Table 1 (5/10) and Figure 2 (17.4%). Here, fewer risk routes are identified for the 2000 scenario as certain European climates in June differ sufficiently from those at SSA airports to be classed as non-suitable. In figure 2, 17.4% of airports included in the analysis have fewer months of suitability when using June as the determining dendrogram threshold month. These are all within the temperate regions of the northern hemisphere.

| Start of | End of | No. changes | No. changes | % airports changed | % airports changed |
| --- | --- | --- | --- | --- | --- |
| season map | season map | to table 1 (/10) | to table 2 (/20) | in figure 2 | in figure 3 |
| Original | Original + 1 month | 0 | 0 | 2.8 | 0.5 |
| Original | Original - 1 month | 0 | 0 | 2.5 | 0.4 |
| Original + 1 month | Original | 0 | 0 | 2.4 | 0.4 |
| Original -1 month | Original | 0 | 0 | 2.8 | 0.4 |
| Original + 1 month | Original + 1 month | 0 | 0 | 3.9 | 0.7 |
| Original - 1 month | Original - 1 month | 0 | 0 | 3.7 | 0.6 |
| Original + 1 month | Original - 1 month | 0 | 0 | 4.1 | 0.8 |
| Original - 1 month | Original + 1 month | 0 | 0 | 4.8 | 0.8 |

Table 1.1. Changes in results following alterations to the malaria seasonality map.

| Dendrogram | No. changes | No. changes | % airports changed | % airports changed |
| --- | --- | --- | --- | --- |
| threshold criteria | to table 1 (/10) | to table 2 (/20) | in figure 2 | in figure 3 |
| a | 0 | 0 | 6.6 | 0 |
| b | 5 | 0 | 17.4 | 0 |
| c | 1 | 0 | 7.8 | 0 |

a: Most climatically dissimilar African airport within its principal transmission season linked to Paris, London or Brussels airports in July and August

b: Most climatically similar African airport within its principal transmission season linked to Paris, London or Brussels airports in June

c: Most climatically similar African airport within its principal transmission season linked to Paris, London or Brussels airports in September

Table 1.2. Changes in results following variation the dendrogram threshold criteria
